# A Novel Gain-Of-Function Mutation of the Proneural *IRX1* and *IRX2* Genes Disrupts Axis Elongation in the Araucana Rumpless Chicken

**DOI:** 10.1371/journal.pone.0112364

**Published:** 2014-11-05

**Authors:** Nowlan H. Freese, Brianna A. Lam, Meg Staton, Allison Scott, Susan C. Chapman

**Affiliations:** 1 Department of Biological Sciences, Clemson University, Clemson, South Carolina, United States of America; 2 Department of Entomology and Plant Pathology, University of Tennessee, Knoxville, Tennessee, United States of America; Instituto Gulbenkian de Ciência, Portugal

## Abstract

Axis elongation of the vertebrate embryo involves the generation of cell lineages from posterior progenitor populations. We investigated the molecular mechanism governing axis elongation in vertebrates using the Araucana rumpless chicken. Araucana embryos exhibit a defect in axis elongation, failing to form the terminal somites and concomitant free caudal vertebrae, pygostyle, and associated tissues of the tail. Through whole genome sequencing of six Araucana we have identified a critical 130 kb region, containing two candidate causative SNPs. Both SNPs are proximal to the *IRX1* and *IRX2* genes, which are required for neural specification. We show that *IRX1* and *IRX2* are both misexpressed within the bipotential chordoneural hinge progenitor population of Araucana embryos. Expression analysis of *BRA* and *TBX6,* required for specification of mesoderm, shows that both are downregulated, whereas *SOX2*, required for neural patterning, is expressed in ectopic epithelial tissue. Finally, we show downregulation of genes required for the protection and maintenance of the tailbud progenitor population from the effects of retinoic acid. Our results support a model where the disruption in balance of mesoderm and neural fate results in early depletion of the progenitor population as excess neural tissue forms at the expense of mesoderm, leading to too few mesoderm cells to form the terminal somites. Together this cascade of events leads to axis truncation.

## Introduction

During secondary body formation the regressing primitive streak and Hensen’s node are transformed into a bulblike structure, the tailbud, a morphologically uniform mass of mesenchyme [1], [2], [3]. The tailbud mesenchyme is located adjacent to the posterior end of the neural tube and notochord, an area known as the chordoneural hinge (CNH) [4], [5]. The CNH together with the dorso-posterior and ventral tailbud populations gives rise to all the derivatives of the tail [6], [7]. These include the precursor to the secondary neural tube (the medullary cord), somite progenitors of the presomitic mesoderm, and the posterior extension of the notochord [3], [4], [6]. The CNH acts as a bipotential population of long-term axial progenitors, contributing cells to both the somitic mesoderm and medullary cord neuroectoderm [6], [7], [8]. The bipotential nature of the CNH requires fine control of expression of neural genes such as *SOX2* and mesodermal genes such as *TBX6* in order to maintain the balance of neural and mesodermal cell fates [9], [10], [11].

Throughout secondary body formation the remaining somites form from the paraxial mesoderm (PM) of the tail [12]. Somites are transitory paired epithelial spheres that differentiate to generate the axial skeleton, including the vertebrae, cartilage, and most of the skeletal musculature and dermis [13]. The number of somites formed is species-specific and highly variable; for example whereas chicken (*Gallus gallus)* has between 51–53 somites, mouse (*Mus musculus)* has approximately 65 somites and the corn snake (*Pantherophis guttatus*) over 300 somites [14], [15]. Somitogenesis is a critical process during axis elongation, and interference brought about by teratogenic factors or the existence of congenital mutations can lead to axis truncation. Many model organisms have been used to study axis elongation including chicken, mouse, and zebrafish [7]. Notably in mice, spontaneous mutants such as the *vestigial tail* mouse, hypomorphic for *Wnt3a*, and the *Brachyury (T)* mouse have been studied as models of axis truncation [16], [17], [18], [19]. Null or reduced expression of either *Wnt3a* or *T* leads to a failure to maintain the CNH progenitor population and failure of mesoderm specification. Additionally, gene knockouts of *FGF8* and *CYP26A1* also result in axis truncation, with the latter required to protect the progenitor population from the apoptotic effects of retinoic acid until extension is complete [20], [21]. Current models of axis length termination include the elimination of the tailbud progenitor population through programmed cell death and diminution of the presomitic mesoderm (PSM) [22], [23], [24], [25]. However, many gaps in knowledge still exist, requiring a better understanding of the genes, signals and regulators of secondary body formation and its subsequent termination.

The Araucana chicken breed has been maintained as show birds for their rumpless (*Rp*) and ear tuft morphology [26], [27], [28], [29], [30]. Rumplessness is an inherited autosomal dominant disorder, which we had previously mapped to a 740 kb region on chromosome 2 [28], [31]. The Araucana model offers an opportunity to further elucidate the morphogenetic and molecular mechanisms required for normal tail development, as well as the cessation of axis elongation, in an accessible model organism. Here, we investigate the mechanism of rumplessness and the identity of the causative mutation.

In the current study we show that misexpression of the *IRX1* and *IRX2* proneural genes, located within our candidate region, precedes a cascade of altered downstream gene expression. This results in a morphogenetic chain reaction including: changes in bipotential progenitor cell fate, premature depletion of progenitors, early termination of somitogenesis, and early apoptosis of the progenitor remnant and posterior axis malformation. Furthermore, we identify two candidate causative mutations, within a narrowed 130 kb region of chromosome 2 through bioinformatics analysis of whole genome resequencing of six Araucana birds. Together, our results provide a greater understanding of the mechanism of secondary body formation, cell fate determination, axial elongation, determination of posterior somite numbers and control of overall tail length.

## Materials and Methods

### Animals

Clemson University IACUC approved the study, protocol number 2011-041. Fertilized chicken eggs were obtained from SkyBlueEgg (Arkansas, U.S.A.) and the Clemson University Poultry Farm. Eggs were incubated at 38.5°C in a humidified chamber to the desired stage. Embryos were staged according to Hamburger and Hamilton (HH) [32]. Skeletal material was the gift of the Araucana Club of America.

### Bone and cartilage staining

Bone and cartilage staining was carried out on E18 Araucana*^Rp^* and tailed controls using Alcian blue (Polysciences) and Alizarin red S (Acros Organics) according to standard procedures [33]. Briefly, Embryos were fixed 3×24 hours in 95% EtOH, 100% EtOH, 2×24 h in 100% Acetone. Cartilage staining (20 mg Alcian Blue in 100 ml of 40% acetic acid glacial/EtOH) was performed from a few hours to overnight depending on sample size. Embryos were rinsed in EtOH for 15 min followed by EtOH for 24 hrs. They were then placed in saturated borax solution 2×24 hours (Na_2_B_4_O_7_10H_2_O in H_2_O). Trypsin solution (0.45 g purified trypsin in 400 mL of 30% borax dissolved in distilled water) at 30°C was used to clear tissue until flesh became translucent and soft (between 1–4 days, depending on size of sample). Alizarin Red S solution (0.5% KOH and 0.1% Alizarin Red S) was used to stain bones (12–24 hours). Samples were then washed in distilled water, followed by a wash in 0.5% KOH solution for 15 min. Excess Alizarin Red S stain was removed using 0.5% KOH solution for 2×24 hours at room temperature under a light source. Samples then went through series of glycerol 0.5% KOH washes (20% glycerol/0.5% KOH, 50/50 and 75/25 mix). Samples were stored in 100% glycerol with 100 mg thymol crystals.

### Somite number counts

Araucana*^Rp^* and controls were incubated to between HH16-25. Embryos were harvested and somite counts performed using a Nikon stereoscopic microscope (control n = 73, Araucana*^Rp^* n = 83). At later stages, between HH22-25, *DACT2* ISH labeling was used to aid counts of the posterior somites. The number of somites in tailed controls was compared against the expected number of somites as described in the normal stage series, and found to match [32].

### Statistical Analysis

Assuming a normal distribution of the data, a two-tailed *t*-test was carried out to test for differences in the average values of samples from experiments for somite counts, proliferation, and TUNEL. Analysis was carried out using Statistical Analysis Software (SAS).

### Immunohistochemistry

Embryos were fixed in 4% paraformaldehyde (PFA/PBS) for 48 hours before being cryoembedded in 15% sucrose/7.5% gelatin/PBS and sectioned on a Leica cryotome at 25 µm. Immunostaining was carried out using our standard protocol [34]. Briefly, sections were blocked in PBS with 0.1% TritonX-100 and 0.2% bovine serum albumin (BSA). Then incubated overnight at 4°C with the primary antibodies anti-E-cadherin (cat 610182, BD Bioscience) and anti-laminin (cat L9393, Sigma). Following washing in PBS sections were incubated at secondary antibodies 1∶200 Alexa Fluor 488 goat anti-mouse IgG and Alexa Fluor 594 goat anti-rabbit IgG (Invitrogen). Following washing in PBS and mounting with SlowFade (Life Technologies), fluorescent images were captured using a Nikon Ti Eclipse confocal microscope.

### In situ hybridization

Whole-mount in situ hybridization (ISH) was performed according to our standard procedures using probes against *BRA* (control = 18, Araucana*^Rp^* n = 14), *CYP26A1* (control = 16, Araucana*^Rp^* n = 8), *DACT2* (control = 19, Araucana*^Rp^* n = 11), *FGF8* (control = 19, Araucana*^Rp^* n = 11), *IRX1* (control = 26, Araucana*^Rp^* n = 12), *IRX2* (control = 25, Araucana*^Rp^* n = 17), *IRX4* (control = 16, Araucana*^Rp^* n = 9), *MESO1* (control = 17, Araucana*^Rp^* n = 9), *RALDH2* (control = 8, Araucana*^Rp^* n = 8), *SOX2* (control = 6, Araucana*^Rp^* n = 9), *TBX6* (control = 29, Araucana*^Rp^* n = 19), and *WNT3A* (control = 18, Araucana*^Rp^* n = 13) [34]. Probes have all been previously described as follows: *DACT2*, *MESO1* and *TBX6* [24], *FGF8* [35], *IRX1*, *IRX2* and *IRX4* [36], *RALDH2* [37] and *WNT3A* [38]. *IRX1, IRX2,* and *IRX4* probes were the generous gift of Dr. Cheryll Tickle. Embryos were cryoembedded in 15% sucrose, 7.5% gelatin/PBS and sectioned on a Leica cryotome. Whole mount embryos and sections were imaged on a Nikon Smz1500 stereomicroscope and Nikon Eclipse 80i compound microscope, respectively using a Qimaging Micropublisher 5.0 camera.

### EdU and TUNEL labeling

For proliferation analysis, Click-iT EdU 488 Imaging Kit (Invitrogen) was used to carry out labeling of cells as previously described [39]. Briefly, embryos were pulsed with EdU for 60 minutes before harvesting and fixation in 4% PFA overnight. Embryos were then cryoembedded in 15%sucrose/7.5%gelatin/PBS and sectioned on a Leica cryotome at 25 µm. Alternating sections were processed for EdU detection with a counterstain of TO-PRO-3 Iodide, or apoptosis detection using the TUNEL method (In Situ Cell Death Detection Kit, TMR red, Roche) with a Hoechst counterstain, and imaged on a Nikon Ti Eclipse confocal microscope. For each sample, a single matching EdU and TUNEL labeled mediolateral image was selected. Image analysis of EdU, TUNEL, and TO-PRO-3 labeling was carried out using ImageJ (control n = 21, Araucana*^Rp^* n = 17). For individual cell counts of EdU, TO-PRO-3, and TUNEL stained cells, a region of interest was manually selected consisting of the tailbud mesenchyme based on location and morphology, excluding the surrounding ectoderm.

### Whole genome sequencing and bioinformatics

DNA samples for six Araucana were acquired from our previous study [31]. Each of the six samples was sequenced on six lanes with an Illumina HiSeq 2000 sequencer. Average genomic coverage was 27.63x and average number of bases sequenced was 29.009 Giga base pairs (Table S1). Sequence reads were trimmed using Trimmomatic and aligned to the corresponding region previously identified to be associated with the rumpless phenotype on chromosome 2 of the ICGSC Gallus_gallus-4.0/galGal4 build using Bowtie2 applications [40], [41]. The mpileup function of SamTools was used to call variants [42]. The view option of bcftools was used to call the genotype at each variant for each individual bird using the bcftools defaults. Variants that were found to be homozygous in all three homozygous rumpless birds, found to be heterozygous in all two heterozygous birds, and were not found in the homozygous tailed bird were considered fixed in the population and targets for genomic variation that may result in the rumpless phenotype. Identified variants were compared against known variants in the Beijing Genomics Institute (BGI) database, and variants that were previously identified not to be involved in rumplessness were removed [43].

## Results

### Araucana*^Rp^* lack the caudal-most vertebrae

**Figure 1:**
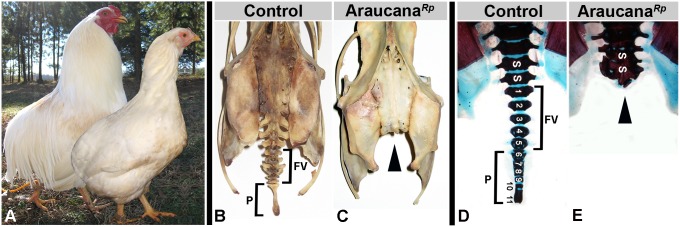
Skeletal analysis shows Araucana*^Rp^* lack caudal vertebrae. Adult Araucana*^Rp^* male and female birds shown in a composite image (A) (courtesy of Fritz Ludwig). Note the characteristic rounded rump, lacking tail structures. Skeletons of control (B) and Araucana*^Rp^* (C) birds (courtesy of the ACA). The free vertebrae and pygostyle are missing in the Araucana*^Rp^* skeleton (arrow). E18 embryos stained with Alcian Blue in control (D) and Araucana*^Rp^* (E). Araucana*^Rp^* embryos lack the free vertebrae and pygostyle. Arrowheads indicate lateral processes. Vertebral elements are numbered from the first free vertebrae (1–5). The more posterior vertebral elements (6–11) fuse to form the mature pygostyle after hatching. FV-free caudal vertebrae, P-pygostyle, S-sacral vertebrae.

The current North American breed standard of the Araucana chicken requires that they lack the caudal vertebrate and other tail structures, appearing rumpless (Fig. 1A). This distinctive morphology is revealed by comparison of adult tailed control and rumpless Araucana (Araucana*^Rp^*) skeletons, where Araucana*^Rp^* lack the free caudal vertebrae and pygostyle of the tail (Fig. 1B–C). By performing cartilage and bone staining (Alcian Blue and Alizarin Red S) from embryonic day E5 to E18, we determined that failure to form the vertebrae, rather than reabsorption occurs. At E18, 5 free caudal vertebrae and 6 fused vertebrae of the pygostyle are observed in controls (Fig. 1D), but are missing in Araucana*^Rp^* (Fig. 1E). From these data we conclude that the rumpless phenotype observed in the Araucana*^Rp^* adult chicken arises during early posterior development due to the lack of free caudal vertebrae and the pygostyle, matching previous observations of rumpless chickens [28].

### Araucana*^Rp^* embryos display truncated tail morphology at the tail organizer stage

**Figure 2:**
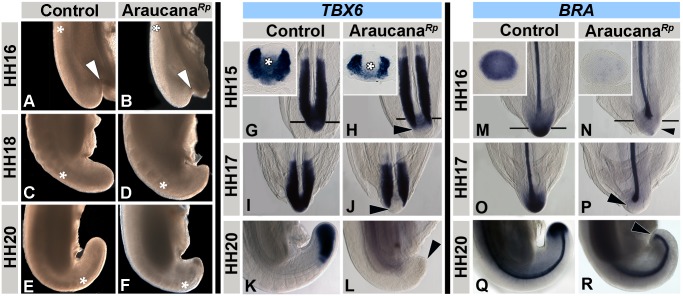
Araucana*^Rp^* embryo tailbud is truncated and downregulates *TBX6 and BRA*. Whole mount tails in lateral view, with posterior at the bottom, dorsal to the left (A–F). At HH16, the angle of tail curvature is narrower in controls (A) compared to Araucana*^Rp^* embryos (B-arrowhead). By HH18, the reduced length and pointed shape of the Araucana*^Rp^* tail is dramatic (D) compared to controls (C). The control tail has curved ventrally by HH20 (E), whereas the Araucana*^Rp^* tail has failed to extend (F). Asterisks denote level of posterior-most somite pair. Somite formation in Araucana*^Rp^* is near the tip of the tail (F-asterisk). Expression patterns of *TBX6* (G–L) and *BRA* (M–R) during tailbud development. ISH expression of *TBX6* in control (G,I,K) and Araucana*^Rp^* (H,J,L) at HH15, 17 and 20. Inset in G and H are transverse sections of respective embryos at level of tailbud, asterisk denotes undifferentiated mesenchyme. Note downregulation of *TBX6* expression in Araucana*^Rp^* (arrowheads) compared to controls. ISH expression of *BRA* in control (M,O,Q) and Araucana*^Rp^* (N,P,R) at HH16, 17, and 20. Inset in M and N are transverse sections of respective embryos at level of tailbud. Note loss of *BRA* expression in tailbud mesenchyme in Araucana*^Rp^* (arrowheads) versus controls.

To examine potential mechanisms resulting in axis truncation, we first determined the earliest stage at which Araucana*^Rp^* embryos displayed morphological differences by examining the gross morphology of the tail region. Formation of the tailbud occurs at HH14-15 and is identical between controls and Araucana*^Rp^* [1], [2], [3]. At HH16, the Araucana*^Rp^* embryo tail appeared less elongated and more pointed than rounded compared to controls (Fig. 2A–B). Moreover, a lesser angle of curvature between the Ventral Ectodermal Ridge (VER) and extraembryonic ectoderm was apparent (arrow, Fig. 2A–B). This became more pronounced at HH18, with reduced distance between the most recently formed somite pair (asterisk) and the end of the tail in Araucana*^Rp^* (Fig. 2C–D). The tip of the tail appeared pointed and the mesenchymal cells underlying the ectoderm cap had dense apoptotic morphology. By HH20 elongation of the tail has ceased in Araucana*^Rp^*, with the most recently formed somite located more posteriorly in the tail than controls (asterisk, Fig. 2E–F).

### Araucana*^Rp^* embryos downregulate *TBX6* and *BRA* at the tail organizer stage

Having identified the critical stage at which morphology was affected we next analyzed *TBX6* and *BRA* expression. Both T-box transcription factors are important for proper axial elongation, with mutations leading to changes in either gene’s expression causing axis truncation [10], [11], [17], [18], [19], [44], [45], [46]. *TBX6* is a marker of the presomitic mesoderm (PSM) and undifferentiated mesenchyme in the tailbud (asterisk, Fig. 2G), acting to indirectly repress the neural transcription factor *SOX2,* in order to specify mesoderm (Fig. 2G,I,K) [9], [11]. Up to HH15 *TBX6* expression was as expected in control and Araucana*^Rp^* embryos. At HH15 (26 pairs of somites), *TBX6* expression was downregulated in the undifferentiated mesenchyme of the Araucana*^Rp^* tailbud (asterisk, Fig. 2H, inset). The tailbud comprises all mesenchyme posterior to the neural tube and notochord, consisting of the chordoneural hinge population (CNH) and tailbud mesenchyme (TBM) that lies directly underneath the ectoderm capping the tail [3], [6]. Expression of *TBX6* was maintained in the PSM in HH15-HH17 embryos, but not the undifferentiated mesenchyme of the TBM (Fig. 2H,J). However, by HH20 *TBX6* expression within the PSM of Araucana*^Rp^* embryos was no longer visible (Fig. 2K,L). This suggests that following downregulation of *TBX6* within the tailbud, Araucana*^Rp^* fail to specify new PSM.

*BRA* is a marker of the TBM and notochord, and is required for mesoderm specification in the tail (Fig. 2M,O,Q) [17], [18], [44]. Heterozygous *T* mutant mice display malformed sacral vertebrae and shortening of the tail due to a failure of axis elongation and somite formation during embryogenesis [18], [19], [44]. Loss of *BRA* expression in Araucana*^Rp^* followed downregulation of *TBX6* within the tailbud mesenchyme at HH16, whereas notochord expression of *BRA* remained unaffected (Fig. 2N,P,R). These results show that the defect leading to Araucana*^Rp^* rumplessness arises early in tailbud development, and involves the downregulation of transcription factors required for specification of the PSM. Observation of the downregulation of *TBX6* and *BRA* within the TBM, which contributes to the PSM, suggests a failure of new PSM specification in Araucana*^Rp^* embryo tails as early as HH15 (26 somites) [6].

### Somitogenesis ends prematurely in Araucana*^Rp^* embryos

**Figure 3:**
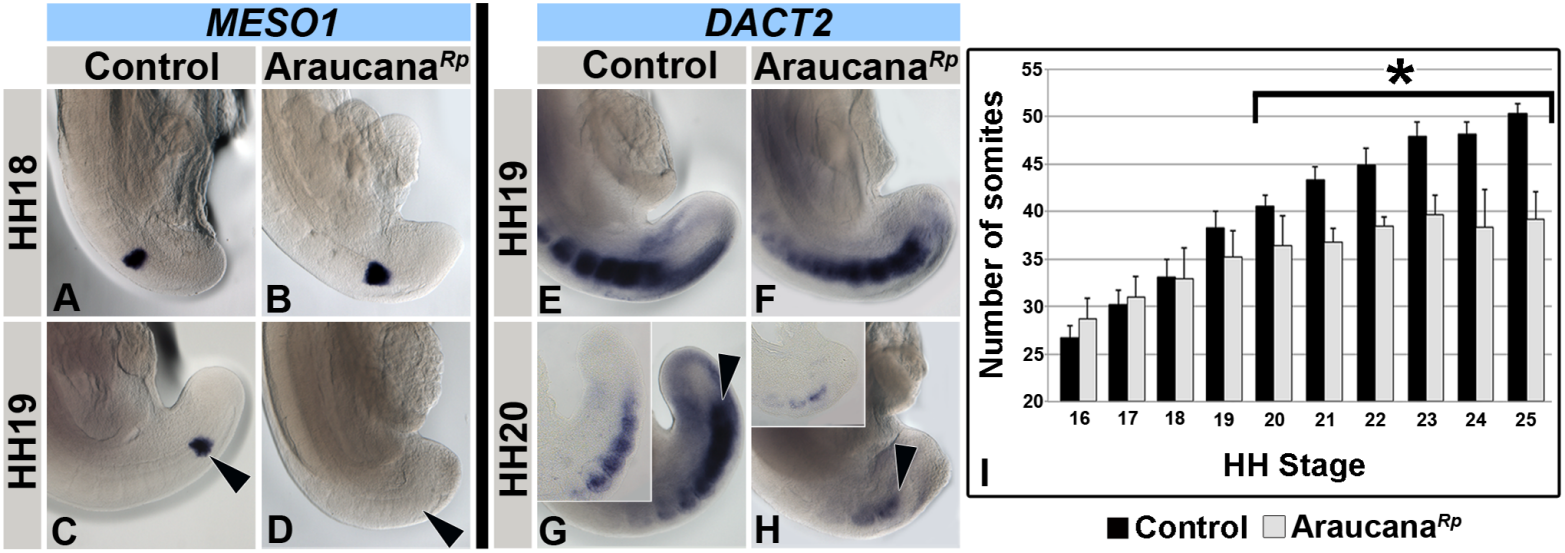
Araucana*^Rp^* form fewer somites. *MESO1* expression in control (A,C) and Araucana*^Rp^* (B,D) embryos at HH18-19. Note downregulation of *MESO1* in HH19 Araucana*^Rp^* (D-arrowhead) compared to controls (C-arrowhead). *DACT2* expression in control (E,G) and Araucana*^Rp^* (F,H) from HH19-20. Inset in G and H are sagittal sections through the paraxial mesoderm from respective embryo. Note decrease in somites labeled by *DACT2* at HH20 in Araucana*^Rp^* (H-arrowhead) compared to control (H-arrowhead). Anterior up in whole mount and section insets. (I) Graph showing number of somites compared to embryonic stage (HH stages 16–25). Araucana*^Rp^* have significantly fewer somites beginning at HH20 (T-test, asterisk marks p<0.01) than controls. Control-Black, Araucana*^Rp^*-Grey.

In tailed chicken embryos axial elongation and somitogenesis continue until HH24-25, when 51–53 somite pairs have formed [24]. Due to the downregulation of *TBX6* and *BRA* and their requirement to specify PSM, we predicted Araucana*^Rp^* would fail to form the correct number of somite pairs [11], [44]. To that end we analyzed *MESO1* expression. *MESO1* is the homolog of mouse *Mesp2*, a transcription factor expressed in the anterior presomitic mesoderm that plays a role in somite segment border formation, and is required for the formation of the next pair of epithelial somites (Fig. 3A,C) [47], [48]. *TBX6* binds to regulatory elements of *Mesp2* and is required for its expression [49]. *MESO1* expression is normally downregulated at HH24-25, marking the end of somitogenesis [24]. We found that *MESO1* expression matched controls (Fig. 3A–B) until HH19, at which point *MESO1* expression was lost in Araucana*^Rp^* (Fig. 3C–D). *MESO1* expression was lost as *TBX6* was downregulated within the remaining PSM in Araucana*^Rp^* (Fig. 2L and 3D). This result suggests that Araucana*^Rp^* somite formation is arrested as early as HH19.

Next we examined expression of the homolog of zebrafish *dpr2*, *DACT2*, a regulator of *WNT* and *TGFβ* signaling that is expressed in the anterior primitive streak, neural crest cells, and most recently formed somites after HH17 [24], [50], [51], [52]. *DACT2* expression in Araucana*^Rp^* labeled a similar number of recently formed somites when compared to controls at HH19 (Fig. 3E–F). However, the distance from the most posterior somite to the tip of the tail was shortened, suggesting less PSM in Araucana*^Rp^* (Fig. 3F). At HH20, *DACT2* labeled fewer somites in Araucana*^Rp^* than controls (Fig. 3G,H). This suggests that somite formation is reduced in HH20 Araucana*^Rp^* as fewer *DACT2* labeled somites equates to a lack of further somite formation. In controls, *DACT2* continued to label recently formed somites through the end of somitogenesis at HH24-25 [24], [52]. The downregulation of *MESO1* and *DACT2* expression 22.5 hours earlier than in controls indicates that the most posterior somites, which give rise to the free caudal vertebrae and pygostyle, fail to form.

To confirm the lack of somite formation we performed somite counts for Araucana*^Rp^* and controls from HH16-25 (Fig. 3I). Beginning at HH20, the number of somites in Araucana*^Rp^* embryos was significantly different than in controls (p<0.01, Fig. 3I). At HH20 Araucana*^Rp^* embryos averaged 4 fewer somites than controls, and by the end of somitogenesis at HH25 Araucana*^Rp^* embryos had on average 11 fewer somites than controls. These data are consistent with our bone and cartilage labeling data in which embryos lacked the 5 free caudal vertebrae and the 6 vertebrae that will later fuse to form the mature pygostyle.

In summary, these data indicate that a mechanism capable of ending somitogenesis prematurely is triggered within Araucana*^Rp^* embryos. The main question that arises from these observations is what is the functional mechanism leading to axial truncation? Several possibilities present themselves, including: changes in proliferation and apoptosis in the CNH population, changes in the balance of cell fates within the bi-potential progenitor population, and changing molecular signaling within the tissues [9], [22], [23], [24], [25]. While none of these is mutually exclusive it is logical to assume that there is a single molecular event that triggers a cascade of downstream changes that eventually results in axial truncation. We next examined each of these possibilities to determine the sequence of events taking place in affected Araucana*^Rp^* embryos.

### *IRX1* and *IRX2* are misexpressed in the Araucana*^Rp^* embryo tailbud

We previously identified a critical region associated with rumplessness in Araucana, containing two genes, *iroquois 1* (*IRX1*) and *iroquois 2* (*IRX2*) [31]. Iroquois-class homeodomain proteins play multiple roles during pattern formation, with one of their primary roles being the initial specification of the vertebrate neuroectoderm [53], [54]. Importantly, *IRX1* and *IRX2* expression is normally excluded from the tailbud. To investigate expression of *IRX1* and *IRX2* in Araucana*^Rp^* embryos we performed in situ hybridization from HH10-HH20.

**Figure 4:**
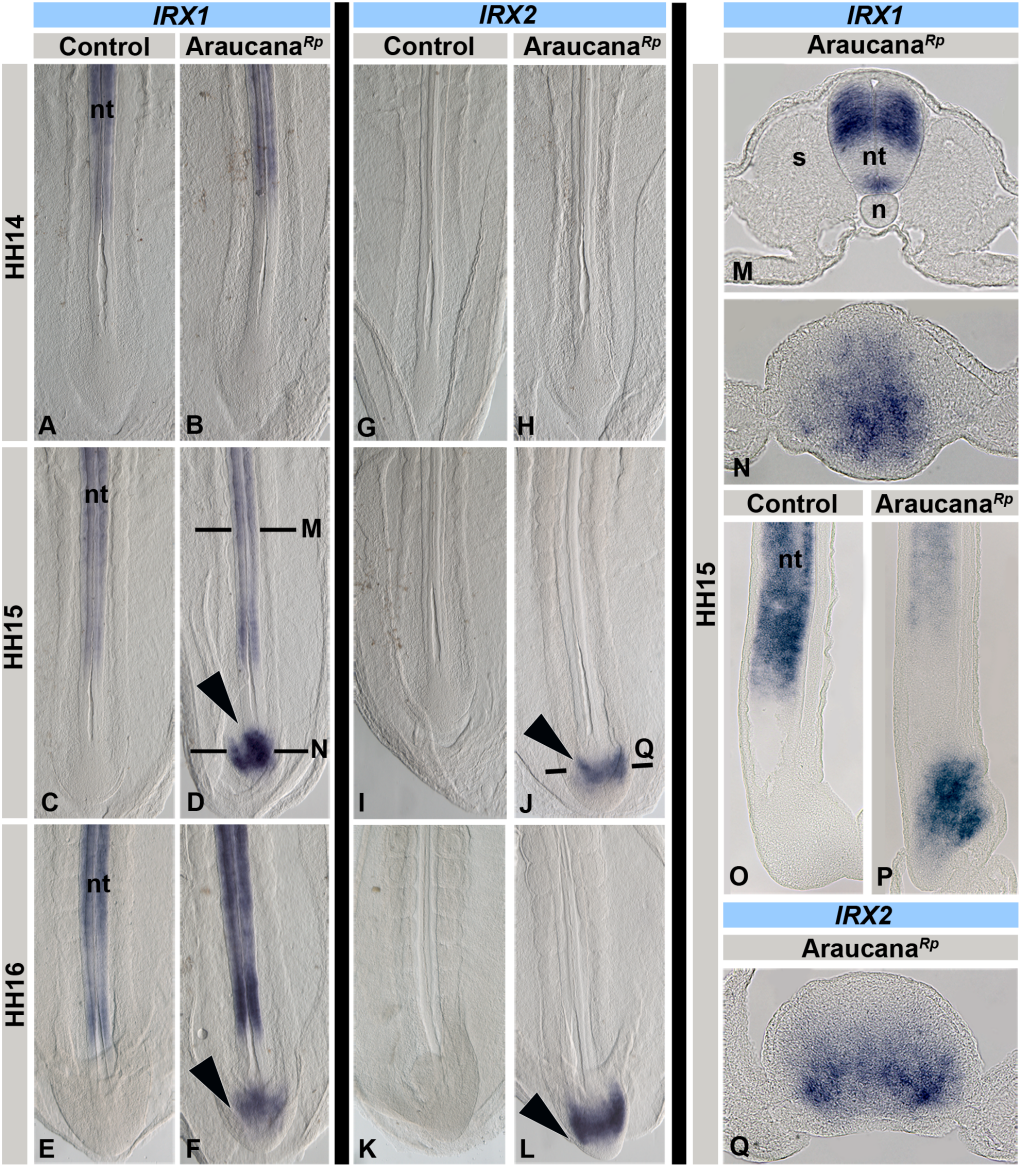
*IRX1* and *IRX2* are misexpressed in Araucana*^Rp^* tailbud. *IRX1* expression pattern (A–F,M–P). (A,C,E) Control embryos express *IRX1* in the neural tube. *IRX1* expression in controls matches Araucana*^Rp^* at HH14 (A,B), but is misexpressed in Araucana*^Rp^* tailbud at HH15 (D-arrowhead). Normal expression at level of somites is within neural tube (transverse section, M). Misexpression in Araucana*^Rp^* can be seen at the level of the chordoneural hinge (transverse section-N and sagittal section-P) compared to expression in HH15 control (sagittal section-O). Misexpression is maintained through HH16 in Araucana*^Rp^* (F-arrow) as compared to control (E). *IRX2* expression pattern (G–L,Q). (G,I,K) Control embryos do not express *IRX2* in the tailbud. No difference in expression between controls and Araucana*^Rp^* seen at HH14 (G,H). *IRX2* is misexpressed in HH15 (J-arrowhead) and HH16 (L-arrowhead) Araucana*^Rp^*. Transverse section of *IRX2* (Q) shows expression similar to *IRX1* at level of chordoneural hinge. A–L - anterior to top. M,N,Q - dorsal to top. O,P - dorsal to left, anterior to top. Abbreviations: nt-neural tube, s-somite, n-notochord.

Expression of *IRX1* in control embryos is restricted to the neural tube of the elongating posterior axis (Fig. 4A,C,E). Analysis of *IRX1* expression up to HH14 revealed identical expression between controls and Araucana*^Rp^* (Fig. 4A–B). At HH15, *IRX1* expression in the neural tube remains unaffected in Araucana*^Rp^* (Fig. 4C–D,M), whereas *IRX1 is* misexpressed medially at the level of the CNH progenitor population (Fig. 4C–D,N–P). *IRX1* misexpression occurred one somite pair earlier than downregulation of *TBX6* expression (Fig. 2H,4D). *IRX1* misexpression in Araucana*^Rp^* tailbud continued at HH16 (Fig. 4E–F) and HH17 (not shown), but by HH18 normal expression was restored (data not shown). These results illustrate a critical time window of *IRX1* misexpression at the onset of tailbud formation and the tail organizer stage.

We then analyzed the expression of *IRX2*, which is also expressed in the neural tube, but restricted more anteriorly than *IRX1* (Fig. 4G,I,K). Analysis of *IRX2* at HH14 revealed no difference in expression between controls and Araucana*^Rp^* (Fig. 4G–H). However, *IRX2* was misexpressed in Araucana*^Rp^* tails beginning at HH15 (Fig. 4I–J). The subpopulation of cells appeared to overlap that of *IRX1* to a limited degree, with a more ventro-lateral subpopulation of mesenchyme labeled (Fig. 4Q). *IRX2* misexpression continues at HH16, but is no longer observed from HH17 (Fig. 4K–L and data not shown).

Although *IRX4* does not fall within the defined critical region, it is within the same genomic cluster as *IRX1* and *IRX2* [31], [55]. *IRX4* expression is restricted primarily to the heart at these stages, suggesting that the regulatory elements controlling its expression are not as closely shared as for *IRX1* and *IRX2* [55], [56], [57], [58]. ISH analysis from HH14-16 revealed identical expression between control and Araucana*^Rp^* embryos (data not shown).

In summary, both *IRX1* and *IRX2* are misexpressed within the Araucana*^Rp^* tailbud at the level of the CNH beginning at HH15. This aberrant expression raises two important questions. First, what is the causative mutation resulting in the gain-of-function of two genes simultaneously? Second, what is the effect of misexpression of two proneural genes within the mesenchyme progenitor population, which commits cells to both mesodermal and neuroectodermal lineages?

### Sequencing of the critical region reveals candidate mutations

**Figure 5:**
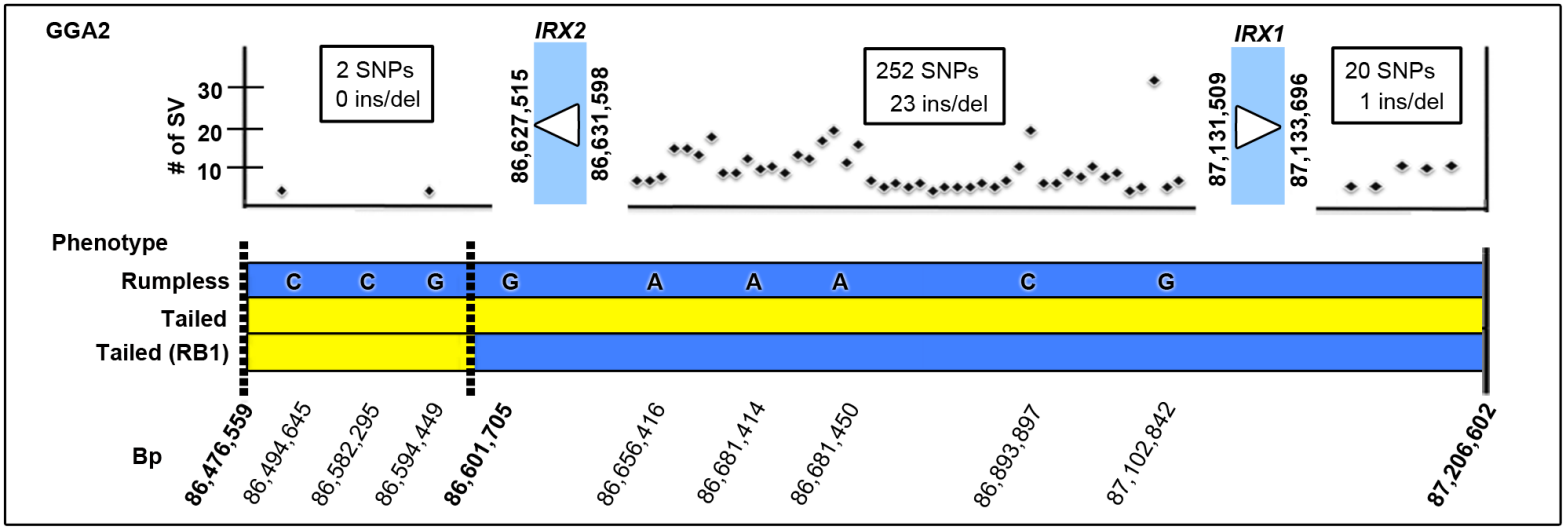
Location of small variants associated with rumplessness in the 740 kb critical region. Top: positional map of the 740 kb critical region located on *Gallus gallus* chromosome 2 (GGA2). Location of *IRX1* and *IRX2* is indicated, with numbers of small variants (SV) (SNPs, insertions and deletions) and their general location and frequency indicated. No small variants were found within *IRX1* or *IRX2* coding sequence. Bottom: relative position of SNPs used to build new critical interval within the established Araucana*^Rp^* haplotype block. Rumpless Araucana*^Rp^* haplotype in blue, with associated SNPs, haplotype from tailed Araucana in yellow. New critical interval defined by RB1 tailed Araucana highlighted by dashed lines.

We previously identified a 740 kb critical region associated with the rumplessness containing both *IRX1* and *IRX2* [31]. In order to identify candidate causative mutations within this region we performed whole genome sequencing on DNA from six Araucana birds. After aligning reads to chicken chromosome 2 (GGA2), small variants (insertions, deletions, and SNPs) were called using the mpileup function of SamTools [42]. Variants were separated into three haplotype groups: three homozygous rumpless, two heterozygous rumpless, and one homozygous tailed Araucana. A total of 2092 unique small variants were identified within the candidate region when compared to the chicken Galgal4 reference sequence. We reduced the list of variants by excluding those that did not hold true to type; keeping only those that occurred in both alleles in the homozygote, a single allele in the heterozygotes, and not present in the tailed Araucana. A total of 316 small variants matched this pattern within the critical region. A further 18 variants that lined up with previously reported variants in tailed birds were also removed [43]. Of the remaining 298 small variants, we identified 274 SNPs and 24 insertion/deletions unique to rumpless Araucana (Fig. 5 and Table S2). None of the identified 298 small variants fell within the sequenced exons or introns of *IRX1* or *IRX2,* suggesting that the causative mutation is within the surrounding regulatory region.

During analysis of the small variants, a promising 55 base pair deletion on chromosome 2 between base pairs 86,830,975–86,831,030 was found to segregate with rumplessness. PCR analysis of the deletion was carried out on 131 Araucana*^Rp^*, 30 tailed Araucana, and 38 non-Araucana tailed birds. In 198 cases, the deletion segregated with the phenotype correctly. However, in one case the genotype of a tailed Araucana male (RB1) did not segregate correctly. Six SNPs throughout the previously identified critical region and three newly identified SNPs from the whole genome sequencing were analyzed to identify the haplotype of RB1 (Fig. 5). RB1 was found to share only part of the rumpless haplotype, delimiting a new critical interval of 125,146 base pairs (86,476,559–86,601,705). Importantly, this interval contains neither *IRX1* nor *IRX2*, indicating that the causative mutation falls outside of either gene’s coding sequence. On analysis of the 298 small variants unique to rumpless Araucana, only two SNPs fall within the new 125 kb critical interval. PCR analysis found both SNPs segregate by phenotype in 6-rumpless Araucana, 6-tailed Araucana, and 5-mixed breed tailed birds. Comparison of the surrounding 300 bp regions of both SNPs revealed that only the region for the SNP at bp location 86,594,449 was conserved in turkey (melGal1), zebra finch (taeGut2), and the medium ground finch (geoFor1). However, further functional testing is required to determine if either of the two identified SNPs within the 125 kb critical region is the heritable cause of rumplessness, however, this is beyond the scope of the current study.

In summary, these results identify two causative candidate SNPs by narrowing down the size of the critical region to 125 kb, as well as supporting the idea that the candidate mutation lies in the flanking regulatory region of *IRX1* and *IRX2* [55].

### Expansion of neural tissue within Araucana*^Rp^* embryo tailbud

**Figure 6:**
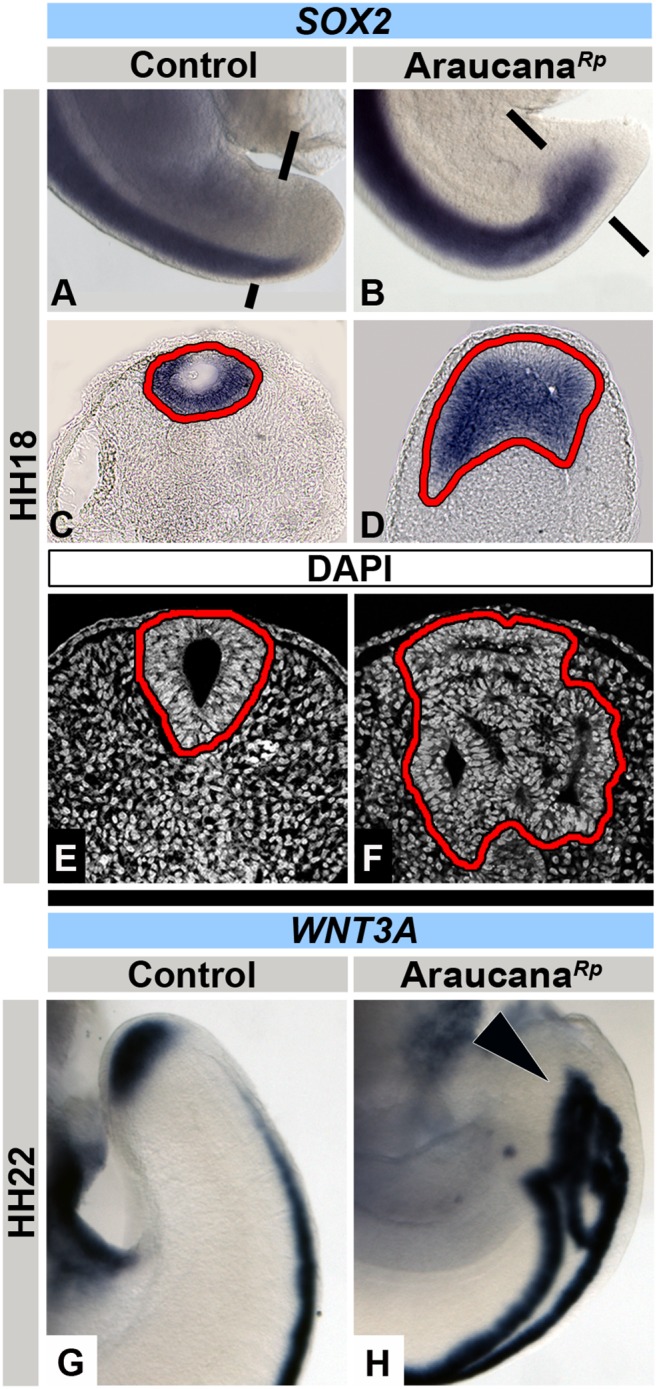
Ectopic neural tissue in Araucana*^Rp^* embryo tailbud. Wholemount images and respective transverse sections showing ISH expression of *SOX2* in control (A,C) and Araucana*^Rp^* (B,D) embryos at HH18. Note the presence of ectopic neural tissue and lumens in Araucana*^Rp^* (highlighted in red) (D) compared to single neural tube and lumen in control (C). DAPI stained transverse sections of control (E) and Araucana*^Rp^* (F) embryo tailbud, highlighting (in red) differences in number of neural tube lumens. Wholemount images of *WNT3A* expression in control (G) and Araucana*^Rp^* (H). Arrowhead denotes ectopic expression of *WNT3A* in ectopic neural tubes of Araucana (H). Anterior to top in wholemount images. Dorsal to top in transverse sections. Sections taken at approximate level of black bars.

*Iroquois* genes are involved in specifying and patterning neural domains [53], [54]. In *Xenopus*, over-expression of *iroquois* causes the neural plate to expand, and promotes the onset of neural differentiation [59], [60]. Importantly, cells of the tailbud have a bipotential fate, becoming mesoderm (paraxial mesoderm) or neuroectoderm (neural tube) [6], [8], [9], [61]. Considering the misexpression of the proneural genes *IRX1* and *IRX2* and the premature downregulation of PSM marker *TBX6*, we predicted that the remaining unspecified progenitor cells would be pushed towards a neural fate. As *TBX6* plays an indirect role in repressing the enhancer of the neural marker *SOX2* we tested the expression of *SOX2* in Araucana*^Rp^* embryos [9], [62]. We found that beginning at HH18, Araucana*^Rp^* embryos displayed ectopic *SOX2* expression, labeling an expanded domain of neural tissue (Fig. 6A–D). In addition, the Araucana*^Rp^* neural tube displayed expanded epithelial structures with multiple open and irregular lumens (Fig. 6E–F). *WNT3A* is expressed in the dorsal midline of the neural tube. At HH22, expression of *WNT3A* in control embryos was limited to the dorsal neural tube, whereas in Araucana*^Rp^* embryos *WNT3A* expression was dramatically altered within the expanded neuroepithelium of the tailbud region (Fig. 6G–H).

Thus, our results demonstrate aberrant neural differentiation in the Araucana*^Rp^* tail as demonstrated by ectopic neural tissue labeled with both *SOX2* and *WNT3A*. This ectopic expression suggests an increase in the number of cells specified to a neural fate. This opens up the question of whether the shift in fate is at the expense of the mesoderm, and therefore the cause of the reduction in PSM in Araucana*^Rp^*.

### Gene expression in the retinoic acid pathway is altered in Araucana*^Rp^* embryos

**Figure 7:**
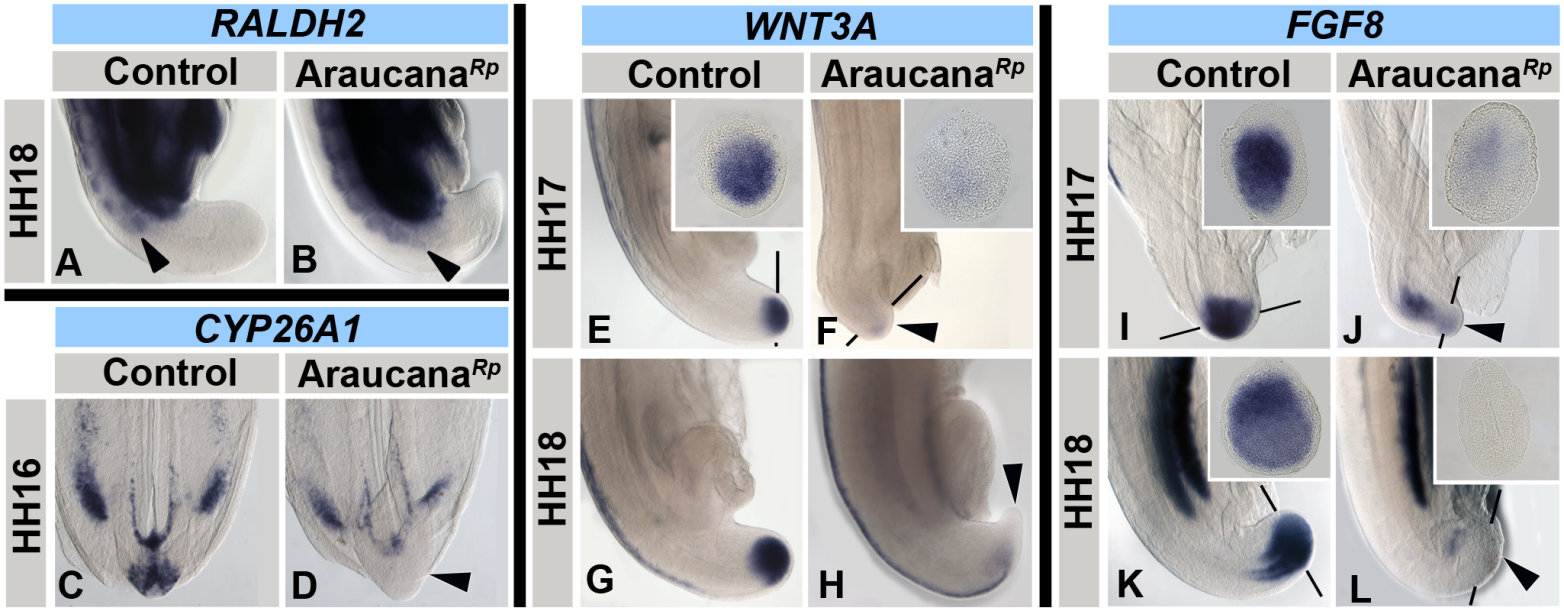
ISH expression pattern of *RALDH2*, *CYP26A1*, *WNT3A* and *FGF8* during tail development. *RALDH2* expression in control (A) and Araucana*^Rp^* (B) embryos at HH18. Note in Araucana*^Rp^* the truncated tail at HH18 coupled with the close expression of *RALDH2* in the formed somites. Posterior-most expression of *RALDH2* is marked with arrowhead. *CYP26A1* expression in control (C) and Araucana*^Rp^* (D) embryos at HH16. Note the lack of expression in Araucana*^Rp^* tailbud (arrowhead). *WNT3A* expression in control (E,G) and Araucana*^Rp^* (F, H) embryos from HH17-18. Note lack of expression in tailbud mesenchyme in Araucana*^Rp^* (arrowheads). *FGF8* expression in control (I,K) and Araucana*^Rp^* (J,L) embryos from HH stages 17–18. Note the downregulation of expression in tailbud beginning at HH17 in Araucana*^Rp^* (arrowheads). Insets are transverse sections at level of tailbud. Anterior is top in whole mount images. Dorsal is top in transverse section insets. Sections were taken at approximate level of black bars.

As the tail elongates and tail mesenchyme cells are displaced anteriorly they are exposed to an increasing concentration of retinoic acid (RA) from the somites [13], [25]. This exposure to RA induces differentiation, as well as plays a role in defining somite segment boundaries at early stages [63], [64]. However, ectopic exposure of the tailbud to RA leads to downregulation of *WNT3A* and *FGF8*, neural expansion, and a decrease in the PSM [24], [25], [65]. Therefore, we evaluated the expression of *RALDH2*, which encodes a dehydrogenase involved in endogenous production of RA [66]. Expression of *RALDH2* in the anterior PSM and the newly formed somites of Araucana*^Rp^* embryos appeared normal compared to the pattern displayed in control embryos at HH18 (Fig. 7A–B). Therefore, there does not appear to be a posterior shift in the production of RA in the tail. However, because of the reduced distance between the rostral PSM and tip of the tail in Araucana*^Rp^* embryos, RA is likely able to influence cells closer to the tip of the tail at earlier stages than normal.

A cytochrome p450 enzyme, *CYP26A1*, is involved in the degradation of RA in the posterior tail, providing a protective effect against RA in the progenitor population during elongation [20], [66], [67]. In *Cyp26a1* ^−/−^ mouse embryos the progenitor population is unprotected against RA and the result is the generation of embryos suffering severe caudal truncation [20]. Therefore, we investigated whether a change in RA degradation could be affecting Araucana*^Rp^* embryos, and found that as early as HH16, *CYP26A1* is downregulated in the Araucana*^Rp^* progenitor mesenchyme (Fig. 7C–D). Thus, although no differences in *RALDH2* expression were observed, lack of *CYP26A1* in Araucana*^Rp^* could lead to increased levels of RA within the progenitor mesenchyme. However, if levels of RA in the tail were higher in Araucana*^Rp^*, we would expect to see a downregulation of *WNT3A* and *FGF8* within the tailbud [16], [24], [25].

*WNT3A* is expressed within the tailbud where it is necessary to specify mesoderm from the bipotential population, and is involved in the proliferation of mesoderm within the tailbud [16], [67], [68], [69], [70]. WNT3A also regulates expression of *FGF8* within the tailbud, where FGF8 acts to maintain expression of *CYP26A1* as well as inhibit expression of *RALDH2*, thereby creating an opposing gradient to RA [64], [71], [72], [73]. This can be disrupted through ectopic exposure to RA in the tailbud, which leads to the downregulation of both *WNT3A* and *FGF8*, and cessation of further axial elongation [24], [25], [65], [74], [75].

We found that until HH16, *WNT3A* expression within the tailbud was indistinguishable between control and Araucana*^Rp^* embryos, but at HH17 (Fig. 7E,F) *WNT3A* expression became significantly downregulated in Araucana*^Rp^* embryos and was lost by HH18 (Fig. 7G,H). We next examined the expression of *FGF8* within the tailbud, where it is expressed throughout tail elongation (Fig. 7I,K). In Araucana*^Rp^*, expression was downregulated as early as HH17 (Fig. 7J), and by HH18 *FGF8* transcripts were undetectable (Fig. 7L).

These data suggest that although there is no change in expression of *RALDH2*, the downregulation of the RA degrading enzyme *CYP26A1* would allow for the exposure of the tailbud to RA. Exposure of the tailbud to RA following the downregulation of *CYP26A1* would explain the subsequent downregulation of *WNT3A* and *FGF8*.

### Araucana*^Rp^* tails have reduced proliferation and increased apoptosis

**Figure 8:**
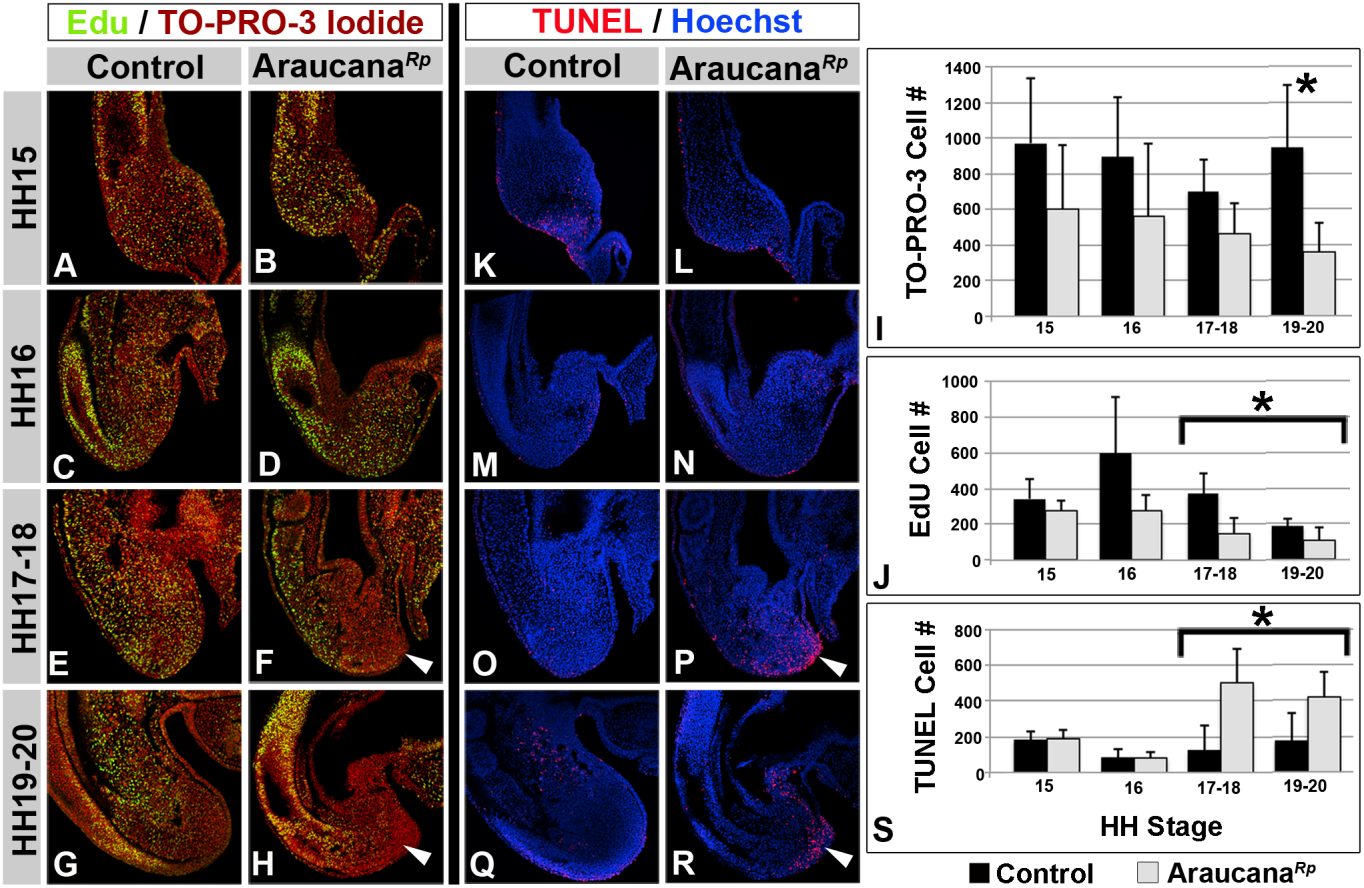
Role of proliferation and apoptosis in Araucana*^Rp^* tailbud development. EdU labeled proliferating cells in tailbud sagittal sections at HH15-20 in controls. (A,C,E,G) and Araucana*^Rp^* (B,D,F,H) embryo tailbud. Green is EdU labeled proliferating cells, red is TO-PRO-3 Iodide labeled nuclei. Arrows denote decreased regions of proliferation in Araucana*^Rp^* compared to controls. (I) Quantification of all TBM cells for control and Araucana*^Rp^* labeled with TO-PRO-3 Iodide. (J) Quantification of proliferating TBM cells for control and Araucana*^Rp^* labeled with EdU. TUNEL labeled apoptotic cells in tailbud sagittal sections from HH stages 15–20 of controls. (K,M,O,Q) and Araucana*^Rp^* (L,N,P,R) embryo tailbud. Red is TUNEL labeled apoptotic cells, blue is Hoechst labeled nuclei. Arrows denote increased areas of apoptosis in Araucana*^Rp^* compared to controls. (S) Quantification of apoptotic TUNEL labeled cells in control and Araucana*^Rp^* TBM. Araucana*^Rp^* have significantly more TUNEL positive cells beginning at HH17 than controls. T-test, asterisk marks p<0.05. Control-Black, Araucana*^Rp^*-Grey. Same embryo (two different sections) was used for both TUNEL and EdU labeling. Anterior is towards the top, dorsal is towards the left in all sections.

The premature downregulation of mesodermal markers *TBX6* and *BRA*, the loss of *WNT3A* which plays a role in proliferation of the progenitor population, and the expanded domain of expression of the neural marker *SOX2* suggests that the progenitor population has been critically jeopardized by forced differentiation towards a neural cell fate. Analysis of total cell numbers by TO-PRO-3 Iodide labeling of the TBM posterior to the hindgut revealed Araucana*^Rp^* have consistently fewer total cells than controls, and this difference was statistically significant by HH19-20 (p<0.05, Fig. 8I). To examine potential drivers of the change in overall cell numbers we compared the levels of proliferation and apoptosis in the tailbud.

Quantification by image analysis showed that proliferation was similar between control and Araucana*^Rp^* TBM up to HH15 (Fig. 8A–B,J). After this a dramatic increase in proliferating cells was noted at HH16 in controls, with less than half the number of proliferating cells in the Araucana*^Rp^* tailbud (Fig. 8C–D,J). From HH17 to HH20 the reduced levels of proliferation are statistically significant (p<0.05, Fig. 8E–H,J). One possible explanation for the reduced rate of proliferation is that the fate change away from mesoderm to neural described previously will reduce the number of progenitor cells as neural cells move out of the cell cycle [76].

Exposure of the tailbud to RA leads to increased levels of apoptosis [25], [65]. Historical studies in rumpless chickens identified degenerating cells within the tailbud and morphologically the tailbud appears to have apoptotic cells in the tip of the tail (Fig. 2D,F) [28]. Therefore, we examined the levels of apoptosis within the tailbud region using TUNEL labeling of sagittal sections. Apoptosis was similar up to HH16 between control and Araucana*^Rp^* embryos (Fig. 8 K–N,S). At HH17-18, however, there was a dramatic rise in apoptosis within the tailbud of Araucana*^Rp^* embryos compared to controls (Fig. 8O–P,S). The increased Apoptosis in Araucana*^Rp^* occurred within the posterior TBM and surface ectoderm, matching the same area in Araucana*^Rp^* that had decreased proliferation (Fig. 8F,P). By HH19-20, apoptosis had increased in the ventral ectodermal ridge of controls, whereas Araucana*^Rp^* apoptosis was still elevated within the remaining posterior TBM, surface ectoderm, and VER (Fig. 8Q–S). These apoptotic Araucana*^Rp^* cells appear in the same position as cells that would normally be expressing *TBX6* and *WNT3A* (Fig. 8P,R). Interestingly, the pattern of apoptosis seen in Araucana*^Rp^* matches that observed by Tenin and coworkers in chicken embryo tailbud at HH26, the end of somitogenesis [24].

## Discussion

The Araucana rumpless phenotype results from a failure to form the most posterior axial somites. Two candidate mutations proximal to the genes *IRX1* and *IRX2* have been identified as associated with the rumpless phenotype. Araucana*^Rp^* exhibit a gain-of-function of both *IRX1* and *IRX2* genes within the tailbud during the tail organizer stage. This gain-of-function precedes observed changes in mesoderm and neural cell specification, maintenance and proliferation of the progenitor population, and regulation of the RA pathway.

Our results indicate that the Araucana*^Rp^* causative mutation is one of two (or both) SNPs within the proximal regulatory region of *IRX1* and *IRX2.* Tena and colleagues found that the 3D architecture of *IRX1* and *IRX2* bring the promoters into close proximity allowing them to share enhancers [55]. The dual misexpression of *IRX1* and *IRX2* supports this co-regulation model in which the Araucana*^Rp^* phenotype results from a gain-of-function mutation in the regulatory region of the *IRX1* and *IRX2* genes. Until further functional testing is done the nature of the causative mutation is speculative, but a mutation could act by removing regionally specific repression/silencing of *IRX1* and *IRX2* within the tailbud, or by creating a new CNH specific enhancer. Importantly, the misexpression of *IRX1* and *IRX2* only occurs post gastrulation at the beginning of the tailbud stages, indicating that the upstream trigger may hold a key to understanding the change between primary and secondary body formation.

**Figure 9:**
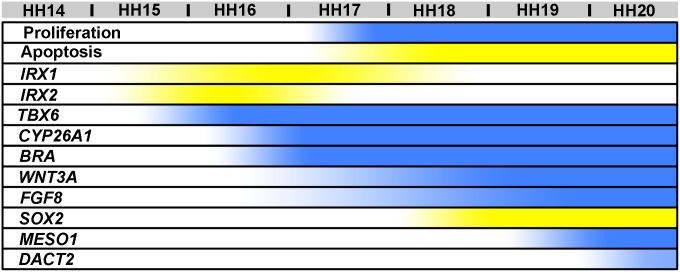
Timeline of changes in proliferation, apoptosis, and gene expression in Araucana*^Rp^*. Visual timeline summarizing changes in proliferation, apoptosis, and gene expression within Araucana*^Rp^* embryo tail compared to control embryos. Yellow is increased expression/prevalence, and blue is decreased expression/prevalence. The *WNT3A* summary represents change in expression observed in posterior tailbud, not neural tube.

The misexpression of *IRX1* and *IRX2* within the tailbud precedes all observed genetic and morphological changes. The *iroquois* genes encode homeodomain-containing transcription factors within the TALE (three amino acid loop extension) family and are involved in proneural fate and patterning through transcriptional repression of neural antagonists [53], [60], [77], [78], [79]. Given the role of *iroquois* genes in specifying neural identity, we hypothesized that *IRX* gene misexpression at the level of the CNH, which is bipotential in chickens, and in the tailbud mesenchyme, a population that has been shown to be bipotential in other vertebrate models including mice and zebrafish, would disrupt the delicate balance between progenitor cell maintenance and mesoderm/neural specification, potentially through the repression of neural antagonists such as *TBX6* [6], [8], [9], [10], [25], [70]. Indeed, immediately upon misexpression of *IRX1* and *IRX2* there is a cascade of gene disruption; immediate downregulation of *TBX6* quickly followed by downregulation of *BRA,* indicating loss of mesoderm identity and an arrest in mesoderm specification [9], [10], [11], [17], [19], loss of *CYP26A1* that is required to degrade RA (protecting progenitor cells from differentiation and apoptosis) [20], [66], [67], upregulation of the neural marker *SOX2* [9], and concomitant loss of *WNT3A* and *FGF8* that are required for maintenance and proliferation of the progenitor population (Fig. 9) [16], [21], [64], [68], [69], [72]. Therefore, we propose a model where proneural gene misexpression overrides the balance of factors within the bipotential progenitor population, steering presumptive mesoderm cells toward the neural lineage.

Morphological changes in the shape of the Araucana*^Rp^* embryo tail are evident as early as HH16 and coincide with the downregulation of the mesoderm markers *TBX6* and *BRA (T* in mice*),* the loss of which lead to axial truncation [11], [17]. *Tbx6* knockout in mice leads to a loss of mesoderm specification, and the upregulation of the neural marker *Sox2* within ectopic neural tubes [10], [11]. *Tbx6* indirectly regulates *Sox2* through repression of its N1 enhancer, making it both necessary to repress neural fate as well as push cells towards a mesoderm fate [9]. The downregulation of *TBX6* expression in Araucana*^Rp^* is the first observed change in gene expression following the misexpression of *IRX1* and *IRX2*. *TBX6* expression is initially lost within the tailbud, not affecting more anterior, previously specified *TBX6*-positive cells. At the anterior boundary of the PSM the cyclic formation of somites continued unaffected, consuming the specified PSM. When these remaining *TBX6* positive cells were incorporated into the most recent somite, somitogenesis arrested prematurely as no further PSM has been specified. *BRA* is required for proper mesoderm cell fate and in its absence embryos fail to form the proper mesoderm structures [18], [80], [81], [82]. *T* homozygote knockout mice embryos fail to form both somites and the notochord, with heterozygotes forming a truncated axis [18], [81]. In Araucana*^Rp^* embryos, notochord expression is maintained, but is absent from tailbud cells by HH17. Interestingly, the downregulation of *BRA* in Araucana*^Rp^* resembles zebrafish *no-tail* mutants (*ntl*, T ortholog), as well as zebrafish treated with RA, which downregulates *ntl* expression [70], [82]. Importantly, as *ntl* both activates *cyp26a1* and functions within an autoregulatory loop with *wnt*, the loss of expression of any of the three genes is sufficient to cause axis truncation [82], [83]. Similarly, the loss of expression of *BRA* in Araucana*^Rp^* could explain the downregulation of *CYP26A1* observed at HH16, as well as the downregulation of *WNT3A* at HH17. Without *TBX6* to repress a neural fate in Araucana*^Rp^* embryos, as well as both *TBX6* and *BRA* to promote a presomitic mesoderm fate, cells that would normally form PSM instead form ectopic neural tissue, or remain in an undifferentiated transition state. Neural cells then leave the progenitor population to join the secondary neural tube (medullary cord), reducing the available progenitor pool and starving the paraxial mesodermal of additional cells required for ongoing somite production.

During somitogenesis, the posterior region of the vertebrate embryo including the PSM is exposed to RA expressed from the somites and *FGF8* expressed from the tailbud, generating two opposing gradients [64], [66], [84], [85], [86]. The tailbud progenitor population is protected from the effects of RA by the action of *CYP26A1*, which metabolizes RA [20], [66]. One of the first events following *IRX1*/*IRX2* misexpression is the loss of *CYP26A1* within the tailbud, though whether the downregulation of *CYP26A1* is a direct consequence of the ectopic *IRX1/IRX2* expression is still unknown. In the tailbud of Araucana*^Rp^*, without the protection of *CYP26A1* we would predict that the levels of RA would be increased, although we have not directly shown this. However, data from chicken and mice studies show that ectopic RA leads to the downregulation of *WNT3A* and *FGF8*, axis truncation, and ectopic neural tissue, which is precisely what we observed in Araucana*^Rp^* embryos [24], [25], [64], [65], [74].

Based on our results changes in proliferation and apoptosis occur following the initial morphological changes of Araucana*^Rp^* embryos at HH16. As cells are constantly moving from the TBM to form the PSM and then somites, a constant supply of new cells is required. Controls display a spike in proliferation at HH16, followed by a declining trend in proliferation. The number of TBM cells as labeled by TO-PRO-3 Iodide in controls appears relatively stable through these stages, indicating that proliferation may not be the only contributor to the cells of the TBM. Cells that migrate through the ventral ectodermal ridge into the TBM are most likely helping to maintain the TBM population in controls [22]. Araucana*^Rp^* do not display the spike in proliferation seen in controls at HH16, with the number of proliferating cells continuously decreasing. A significant decrease in proliferation within the TBM occurs in Araucana*^Rp^* at HH17-18, which coincides spatiotemporally with the downregulation of *FGF8* and *WNT3A*. We are unable to determine from these results whether the downregulation of *FGF8* and/or *WNT3A* directly causes the decrease in proliferation. However, studies in mice have shown that *Wnt3a* plays a role in the generation and proliferation of cells within the tailbud, and loss of *Wnt3a* signaling leads to axis truncation and formation of ectopic neural tissue [16], [68], [69]. This suggests that *WNT3A* is required for proper patterning of presumptive presomitic mesoderm cells and without *WNT3A* there is an expansion of neural tissue, as observed by the ectopic *SOX2*-positive neural tubes in Araucana*^Rp^* embryos [10], [69]. Furthermore, as cells within the TBM of Araucana*^Rp^* are pushed towards a neural fate, they would exit the cell cycle ceasing proliferation [76]. As Araucana*^Rp^* display ectopic neural tissue by HH18, cell cycle exiting due to neural differentiation could also explain the differences in proliferation observed. With the decrease in proliferation within the TBM relative to controls, Araucana*^Rp^* have fewer overall TBM cells, and are unable to produce additional PSM.

Apoptosis occurs within the tailbud of control embryos and by the end of somitogenesis (HH26) is localized to the posterior-most TBM and surface ectoderm [23], [24], [87]. Tenin and coworkers proposed that the localized apoptosis in the posterior TBM contributes to the removal of the remaining progenitor cells [24]. The increase and localization of apoptosis in Araucana*^Rp^* beginning at HH17-18 mirrors that seen in controls at HH26. In both controls and Araucana*^Rp^* this coincides with the downregulation of *CYP26A1, FGF8*, and *WNT3A* expression. Furthermore, exposure to ectopic RA leads to downregulation of *WNT3A* and apoptosis in the tailbud [24], [65], [74]. It is therefore likely that in Araucana*^Rp^* embryos the apoptotic event that is triggered at HH17-18 results from exposure to increased levels of RA within the tailbud. Thus, changes in both apoptosis and proliferation do not appear to be the initial cause of changes in the morphology of the Araucana*^Rp^* phenotype, but rather occur following the initial changes in gene expression within the TBM. As proliferation decreases, and apoptosis increases, the diminution of the TBM ensues, leading to a diminished progenitor population in Araucana*^Rp^* embryos by HH19-20. As cells from this progenitor pool populate both the neural tube and PSM, Araucana*^Rp^* are unable to contribute enough cells to the PSM to continue axis elongation, as there is both a decrease in the number of progenitor cells, and an increase in cells forming neural tissue.

In conclusion, we have provided evidence that a novel gain of function is responsible for the Araucana rumpless phenotype. Hence, the Araucana rumpless mutation (*Rp*) is separate from the spontaneous mutations that cause the lack of a tail in chickens [88]. This study has highlighted the fine control required to maintain axis elongation and has added additional evidence that avian tailbud cells are bipotential, continuing to make germ layer decisions between neural and mesoderm post gastrulation similar to mice and zebrafish [8], [70]. Considering that mutants such as *T* and *Wnt3a* mice are a loss of function, it was surprising that a similar phenotype occurred in Araucana as a gain of function. These results provide insight into a novel developmental mechanism controlling the termination of axis elongation and therefore total axis length. Future studies are required to better understand how the Araucana gain of function mutation drives expression to unbalance the bipotential fate, and whether the same mechanism can control somite numbers in other organisms.

## Supporting Information

Table S1 **Number of reads, bases, coverage, SNPs, and INDELS for each Araucana following WGS.**
(DOCX)

Table S2 **Complete list of 298 unique small variants found from WGS.**
(DOCX)
