# Supplementary material for: A Novel Gain-Of-Function Mutation of the Proneural IRX1 and IRX2 Genes Disrupts Axis Elongation in the Araucana Rumpless Chicken
Source: PLoS One. 2014 Nov 5;9(11):e112364. doi: 10.1371/journal.pone.0112364 (PMC4221472; doi:10.1371/journal.pone.0112364)
Supplement: Table S1 — Number of reads, bases, coverage, SNPs, and INDELS for each Araucana following WGS. (DOCX) [file pone.0112364.s001.docx]

| **Sample** | **# Reads** | **# Bases (bp)** | **Coverage** | **# SNPs** | **# INDELS** |
| --- | --- | --- | --- | --- | --- |
| **Homo-Rumpless (32)** | 246,487,326 | 23,772,362,110 | 22.64 | 723 | 188 |
| **Homo-Rumpless (33)** | 339,674,102 | 36,474,356,076 | 34.74 | 750 | 181 |
| **Homo-Rumpless (38)** | 350,207,902 | 33,513,411,752 | 31,92 | 751 | 176 |
| **Hetero-Rumpless (39)** | 235,911,062 | 22,863,519,234 | 21.77 | 1018 | 51 |
| **Hetero-Rumpless (59)** | 243,759,626 | 23,629,533,581 | 22.50 | 1434 | 116 |
| **Homo-Tailed (49)** | 348,219,696 | 33,805,751,523 | 32.20 | 466 | 102 |
| **Average** | 294,043,286 | 29,009,822,379 | 27.63 | 857 | 136 |
