# Supplementary material for: A Novel Gain-Of-Function Mutation of the Proneural IRX1 and IRX2 Genes Disrupts Axis Elongation in the Araucana Rumpless Chicken
Source: PLoS One. 2014 Nov 5;9(11):e112364. doi: 10.1371/journal.pone.0112364 (PMC4221472; doi:10.1371/journal.pone.0112364)
Supplement: Table S2 — Complete list of 298 unique small variants found from WGS. (DOCX) [file pone.0112364.s002.docx]

| **Position: Gga2:** | **Reference** | **Alternative** | **Insertion/Deletion** |
| --- | --- | --- | --- |
| 86494645 | A | C |  |
| 86594449 | A | C |  |
| 86681450 | C | A |  |
| 86684328 | G | A |  |
| 86684477 | G | T |  |
| 86685406 | A | G |  |
| 86686422 | G | A |  |
| 86687620 | T | C |  |
| 86698409 | C | T |  |
| 86699128 | A | G |  |
| 86704174 | G | A,T |  |
| 86704213 | G | A |  |
| 86705985 | C | T |  |
| 86706502 | C | T |  |
| 86706842 | G | A |  |
| 86707594 | T | A |  |
| 86708924 | G | A |  |
| 86709322 | T | A |  |
| 86712359 | C | A |  |
| 86712610 | G | A |  |
| 86712648 | G | A |  |
| 86713857 | T | C |  |
| 86714593 | C | T |  |
| 86714618 | A | C |  |
| 86715129 | G | A |  |
| 86715918 | A | G |  |
| 86716423 | G | C |  |
| 86716426 | T | G |  |
| 86716537 | G | A |  |
| 86717377 | A | C |  |
| 86717397 | G | A |  |
| 86719914 | G | A |  |
| 86720587 | G | A |  |
| 86722215 | G | T |  |
| 86722228 | C | T |  |
| 86724124 | G | C |  |
| 86725703 | T | A |  |
| 86726446 | tgg | tg | DEL |
| 86727746 | C | T |  |
| 86728107 | A | C |  |
| 86729365 | C | T |  |
| 86729937 | T | C |  |
| 86731937 | G | A |  |
| 86733484 | G | A |  |
| 86733659 | A | G |  |
| 86733884 | C | T |  |
| 86736254 | G | A |  |
| 86736376 | C | T |  |
| 86736608 | A | G |  |
| 86737461 | A | G |  |
| 86737625 | A | T |  |
| 86737895 | T | G |  |
| 86738343 | G | T |  |
| 86738396 | T | C |  |
| 86738858 | A | T |  |
| 86739486 | A | C |  |
| 86740083 | T | C |  |
| 86742751 | t | tT | INS |
| 86742940 | G | C |  |
| 86743922 | A | G |  |
| 86743955 | T | C |  |
| 86747675 | G | C |  |
| 86750371 | C | T |  |
| 86751107 | T | G |  |
| 86752817 | C | A |  |
| 86753131 | A | G |  |
| 86755891 | T | G |  |
| 86758150 | T | C |  |
| 86759027 | G | A |  |
| 86759634 | t | tT,tTT | INS |
| 86762754 | G | A |  |
| 86767068 | G | A |  |
| 86768354 | T | C |  |
| 86768703 | A | C |  |
| 86768748 | T | G |  |
| 86769557 | A | G |  |
| 86770533 | A | C |  |
| 86770570 | C | T |  |
| 86771825 | C | G |  |
| 86774117 | T | C |  |
| 86775523 | T | C |  |
| 86778009 | C | T |  |
| 86779849 | A | G |  |
| 86780488 | A | G |  |
| 86782539 | gta | gTAta | INS |
| 86782567 | T | G |  |
| 86786785 | A | G |  |
| 86787122 | G | A |  |
| 86790267 | T | C |  |
| 86790652 | C | A |  |
| 86791233 | C | T |  |
| 86793672 | t | tT | INS |
| 86793892 | G | T |  |
| 86797917 | C | T |  |
| 86798618 | a | aA | INS |
| 86798797 | C | T |  |
| 86803055 | T | C |  |
| 86804507 | A | T |  |
| 86805523 | A | G |  |
| 86808718 | A | G |  |
| 86808724 | A | T |  |
| 86809850 | G | C |  |
| 86810947 | A | G |  |
| 86811657 | T | C |  |
| 86811902 | G | A |  |
| 86812434 | C | G |  |
| 86813441 | G | T |  |
| 86814986 | A | G |  |
| 86815633 | C | G |  |
| 86816161 | A | G |  |
| 86817355 | A | G |  |
| 86819132 | A | G |  |
| 86819934 | taac | t | DEL |
| 86821326 | C | T |  |
| 86822551 | att | at | DEL |
| 86823990 | A | T |  |
| 86824028 | aca | acaGTCA | INS |
| 86825950 | A | T |  |
| 86827415 | C | T |  |
| 86827495 | G | A |  |
| 86827602 | G | A |  |
| 86828215 | t | tT | INS |
| 86829589 | T | G |  |
| 86830468 | A | T |  |
| 86830671 | G | A |  |
| 86831961 | A | C |  |
| 86832195 | G | T |  |
| 86833078 | A | C |  |
| 86833410 | G | A |  |
| 86834760 | C | T |  |
| 86834923 | T | C |  |
| 86835311 | T | C |  |
| 86835329 | G | C |  |
| 86835470 | A | C |  |
| 86835804 | T | C |  |
| 86836009 | G | A |  |
| 86836341 | A | G |  |
| 86836766 | G | T |  |
| 86836780 | G | T |  |
| 86837128 | T | G |  |
| 86837405 | T | C |  |
| 86838428 | C | T |  |
| 86838877 | C | T |  |
| 86839155 | G | A |  |
| 86840709 | C | G |  |
| 86840988 | G | A |  |
| 86842570 | C | A |  |
| 86844979 | C | T |  |
| 86845003 | C | G |  |
| 86845072 | G | A |  |
| 86845644 | G | A |  |
| 86846131 | C | A |  |
| 86846197 | ag | a | DEL |
| 86846762 | A | T |  |
| 86851672 | G | T |  |
| 86852106 | C | G |  |
| 86856411 | G | A |  |
| 86856931 | G | A |  |
| 86857246 | G | A |  |
| 86857342 | A | G |  |
| 86857396 | A | G |  |
| 86858256 | G | C |  |
| 86859851 | agtgt | agt | DEL |
| 86859940 | G | C |  |
| 86862557 | C | G |  |
| 86862694 | T | C |  |
| 86863554 | G | A |  |
| 86864382 | T | C |  |
| 86864705 | A | G |  |
| 86865584 | T | C |  |
| 86868656 | T | G |  |
| 86869057 | C | A |  |
| 86876979 | C | G |  |
| 86892751 | C | T |  |
| 86893790 | C | A |  |
| 86899158 | a | aA | INS |
| 86907665 | A | G |  |
| 86910242 | C | T |  |
| 86932488 | A | G |  |
| 86936953 | A | G |  |
| 86951292 | T | G |  |
| 86958052 | C | T |  |
| 86958746 | T | C |  |
| 86970924 | G | A |  |
| 86980984 | T | G |  |
| 86981130 | A | T |  |
| 86984157 | G | A |  |
| 86988054 | C | T |  |
| 86988275 | A | G |  |
| 86988288 | T | C |  |
| 86990437 | T | C |  |
| 86992548 | ta | tTa,tTTa,t | INS/DEL |
| 86993160 | T | C |  |
| 86994443 | C | T |  |
| 86995937 | A | T |  |
| 86996876 | A | G |  |
| 86999006 | G | A |  |
| 86999366 | A | C |  |
| 86999597 | T | C |  |
| 86999679 | T | C |  |
| 87000060 | T | A |  |
| 87000246 | A | C |  |
| 87000606 | C | A |  |
| 87000669 | A | T |  |
| 87000908 | T | C |  |
| 87001062 | a | aA | INS |
| 87001492 | C | T |  |
| 87001516 | a | aA | INS |
| 87001997 | T | C |  |
| 87003205 | C | T |  |
| 87003250 | A | G |  |
| 87011376 | C | T |  |
| 87012528 | cat | c | DEL |
| 87018516 | caaa | caa | DEL |
| 87021309 | A | G |  |
| 87030530 | A | T,C |  |
| 87031833 | G | A |  |
| 87032892 | A | G |  |
| 87033167 | C | A |  |
| 87034055 | T | C |  |
| 87036590 | T | C |  |
| 87037565 | A | C |  |
| 87042443 | A | G |  |
| 87044047 | T | G |  |
| 87046920 | G | C |  |
| 87048148 | C | T |  |
| 87049332 | C | T |  |
| 87051183 | a | aA,aAA | INS |
| 87051297 | a | aA | INS |
| 87053604 | C | T |  |
| 87054410 | A | T |  |
| 87056366 | C | G |  |
| 87059916 | G | A |  |
| 87063012 | A | G |  |
| 87063169 | T | C |  |
| 87065745 | T | C |  |
| 87067203 | T | G |  |
| 87067365 | A | G |  |
| 87069944 | G | A |  |
| 87073897 | A | G |  |
| 87094774 | C | A |  |
| 87095979 | A | G |  |
| 87096283 | T | A |  |
| 87096325 | A | T |  |
| 87096480 | gccc | gcc | DEL |
| 87096759 | T | A |  |
| 87096989 | G | T |  |
| 87097004 | C | T |  |
| 87097634 | A | G |  |
| 87097705 | C | T |  |
| 87097842 | C | T |  |
| 87098932 | T | A |  |
| 87098990 | T | C |  |
| 87099124 | C | A |  |
| 87099209 | G | A |  |
| 87099217 | A | C |  |
| 87099270 | G | A |  |
| 87099279 | A | T |  |
| 87099296 | T | C |  |
| 87099490 | G | A |  |
| 87099639 | C | T |  |
| 87099846 | C | T |  |
| 87100156 | G | A |  |
| 87100540 | C | T |  |
| 87101033 | tgg | tAAgg | INS |
| 87101060 | A | G |  |
| 87102025 | A | G |  |
| 87103001 | T | C |  |
| 87103134 | A | C |  |
| 87103162 | G | A |  |
| 87103457 | A | G |  |
| 87103708 | G | C |  |
| 87112798 | A | T |  |
| 87121387 | a | aA,aAA | INS |
| 87121472 | T | C |  |
| 87123513 | G | A |  |
| 87125327 | G | A |  |
| 87156231 | T | A |  |
| 87174243 | taagaaa | taa | DEL |
| 87176155 | G | A |  |
| 87176736 | C | T |  |
| 87177011 | T | A |  |
| 87178003 | G | A |  |
| 87179604 | T | G |  |
| 87182967 | A | G |  |
| 87183517 | C | A |  |
| 87186720 | A | G |  |
| 87188693 | A | C |  |
| 87189218 | C | A |  |
| 87191668 | A | T |  |
| 87192961 | G | A |  |
| 87194100 | T | A |  |
| 87195847 | G | A |  |
| 87196604 | G | T |  |
| 87196624 | T | A |  |
| 87201677 | G | T |  |
| 87201941 | A | C |  |
| 87203123 | T | A |  |
